# The Absence of Thyroid-Stimulating Hormone Receptor Expression on Natural Killer T Cells: Implications for the Immune–Endocrine Interaction

**DOI:** 10.3390/ijms252111434

**Published:** 2024-10-24

**Authors:** Emilia Adamska-Fita, Przemysław Wiktor Śliwka, Małgorzata Karbownik-Lewińska, Andrzej Lewiński, Magdalena Stasiak

**Affiliations:** 1 Department of Endocrinology and Metabolic Diseases, Polish Mother’s Memorial Hospital—Research Institute, 93-338 Lodz, Poland; emila0079@gmail.com (E.A.-F.); p.sliwka87@gmail.com (P.W.Ś.); malgorzata.karbownik-lewinska@umed.lodz.pl (M.K.-L.); andrzej.lewinski@umed.lodz.pl (A.L.); 2 Department of Endocrinology and Metabolic Diseases, Medical University of Lodz, 93-338 Lodz, Poland

**Keywords:** natural killer T cells, thyroid-stimulating hormone receptor, autoimmune thyroid disease

## Abstract

The expression of thyroid-stimulating hormone receptor (TSHR) has been documented on various immune cells, including B lymphocytes, T lymphocytes, Natural Killer (NK) cells, monocytes, and dendritic cells (DCs). Natural Killer T (NKT) cells serve as a crucial link between innate and adaptive immunity, playing significant roles in immunological interactions and autoimmune diseases. The aim of the present study was to evaluate the presence of TSHR on NKT cells. Our research involved patients with thyroid disease, as well as healthy controls. Peripheral blood mononuclear cells (PBMCs) and, thereafter, NKT cells were isolated from 86 patients with benign nodular thyroid disease with and without autoimmune thyroid disease (AITD) (28 and 56 cases, respectively), and TSHR expression was analyzed using fluorescence-activated cell sorting (FACS). In order to confirm the results, the reverse-transcription polymerase chain reaction (RT-PCR) method was used in cells obtained from healthy individuals. Our findings obtained with application of the FACS method revealed that TSHR is not expressed on NKT cells in either AITD or non-AITD patients, though TSHR was detected in the total PBMC population (TSHR+ cells 2.77%). The absence of TSHR on NKT cells was further confirmed with RT-PCR in healthy individuals (*p* < 0.0001). These results questioned the previously suggested direct influence of NKT cells on AITD development.

## 1. Introduction

The functional interplay between the immune and endocrine systems is complex, with numerous cell types and humoral mediators involved, yet not fully understood [1]. Apart from the well-known direct effects of thyroid hormones on the immune system, alterations in the hypothalamic–pituitary–thyroid axis, particularly changes in thyroid-stimulating hormone (TSH) levels, may be of significance. While the role of thyroid-stimulating hormone receptor (TSHR) in thyroid function is well established, recent studies suggested TSHR expression in certain immune cells, potentially influencing immune responses [2,3]. In studies performed by immunoprecipitation and by flow cytometry, TSHRs were found on B and T lymphocytes, Natural Killer (NK) cells, monocytes, and—in a large amount—on dendritic cells (DCs) [2,3]. Although the impact of TSH levels on the quantity of Natural Killer T (NKT) cells in peripheral blood has been postulated [4,5], no reported studies have explored the expression of TSHR on NKT cells.

NKT cells represent a unique T cell subpopulation characterized by the presence of markers of both NK cells (NK1.1, CD56) and T cells (TCR—T cell receptor). This dual marker expression makes NKT cells essential for bridging innate and adaptive immunity. NKT cells constitute approximately 0.1% of peripheral blood lymphocytes [6]. Unlike conventional T αβ lymphocytes, NKT cells possess TCR receptors capable of recognizing glycolipid, glycosphingolipid, and lipid structures presented on non-polymorphic CD1d molecules on both professional and non-professional antigen-presenting cells. This unique feature allows NKT cells to recognize both foreign and self-lipid antigens, granting them regulatory and potentially effector roles in various immune responses, including those related to autoimmune diseases, allergies, infections, and cancers [7].

NKT cells are crucial regulators of autoimmune diseases [8,9]. Dysregulation or deficiency of NKT cells is associated with type 1 diabetes, lupus erythematosus, multiple sclerosis, myasthenia gravis, Guillain–Barré syndrome, and autoimmune thyroid diseases (AITDs) [10,11,12,13,14]. AITDs are a group of disorders characterized by an immune system attack against the thyroid gland antigens, including Graves’ disease (GD) and Hashimoto’s thyroiditis (HT). These diseases involve complex interactions between genetic predispositions and environmental factors, resulting in autoantibody production targeting thyroid-specific proteins, including TSHR [15]. TSHR is part of the G protein-coupled receptor group and is composed of a wide extracellular domain, seven transmembrane passages, and a small intracellular domain [16]. TSHR is mostly expressed in the basolateral membrane of thyrocytes where its stimulation enhances iodine uptake, the synthesis and secretion of thyroid hormones, and the proliferation of thyroid follicular cells, and regulates the expression of thyroid-specific genes like those coding thyroglobulin (Tg), thyroid peroxidase (TPO), and sodium/iodide symporter (NIS) [17,18,19,20]. Moreover, several extrathyroidal cell types with expression of TSHR have been revealed, including immune system cells [2,3,21]. Regardless of the low TSHR expression on non-thyroidal cells, the very high binding affinity for TSH contributes to activating the response despite the low density of TSHR on the cellular surface [22]. Many studies have been conducted to test molecular mechanisms involving TSHR in the pathogenesis of AITD. Cuddihy et al. in 1995 discovered the first potential single-nucleotide polymorphism (SNP) of codon 52 of TSHR gene connected with GD in a female population [23]. Recent studies, including three meta-analyses, confirmed important associations between SNPs in intron 1 of the TSHR gene and the risk of GD [24,25,26]. In a previous study performed in our center, a significant increase in the percentage of NKT cells after administration of recombinant human TSH (rhTSH) was observed [4], suggesting a direct mechanism of TSH action on NKT cells. Such a mechanism was also postulated in other studies [5]. Therefore, in order to evaluate whether the mechanism of TSH action on NKT is direct or indirect, the present study aimed to assess TSHR expression on NKT cells, analyzing NKT cells isolated from the peripheral blood of individuals with and without AITDs.

## 2. Results

### 2.1. Comparison of AITD and Non-AITD Groups

The mean age of the patients was 55.54 ± 11.4 and 61.72 ± 14.43 years in the AITD and non-AITD groups, respectively. The non-AITD group included 45 women and 13 men, while the AITD group consisted of 23 women and 5 men, with 2 cases of overt hyperthyroidism, 3 cases of subclinical hyperthyroidism, and 2 cases of subclinical hypothyroidism.

The AITD and non-AITD groups were similar in terms of age and gender. The comparison of both groups revealed statistically significant higher anti-TPO and anti-Tg levels in patients with AITD. TRAb level was higher in the AITD group but the difference did not reach statistical significance, as there was only one patient with newly diagnosed GD and a high TRAb level, while other GD patients were either on thiamazole or had received radioiodine therapy. The characteristics of the AITD and non-AITD groups are presented in Table 1.

### 2.2. Absence of TSHR Expression on NKT Cells

No expression of TSHR on the surface of NKT cells was detected by FACS in either the AITD or non-AITD patient group. Neither thyroid function nor thyroid-related pharmacotherapy in AITD group influenced this observation. This finding was further confirmed by an analysis performed with RT-PCR gene assays in the cells obtained from the healthy blood donors.

**Figure 1:**
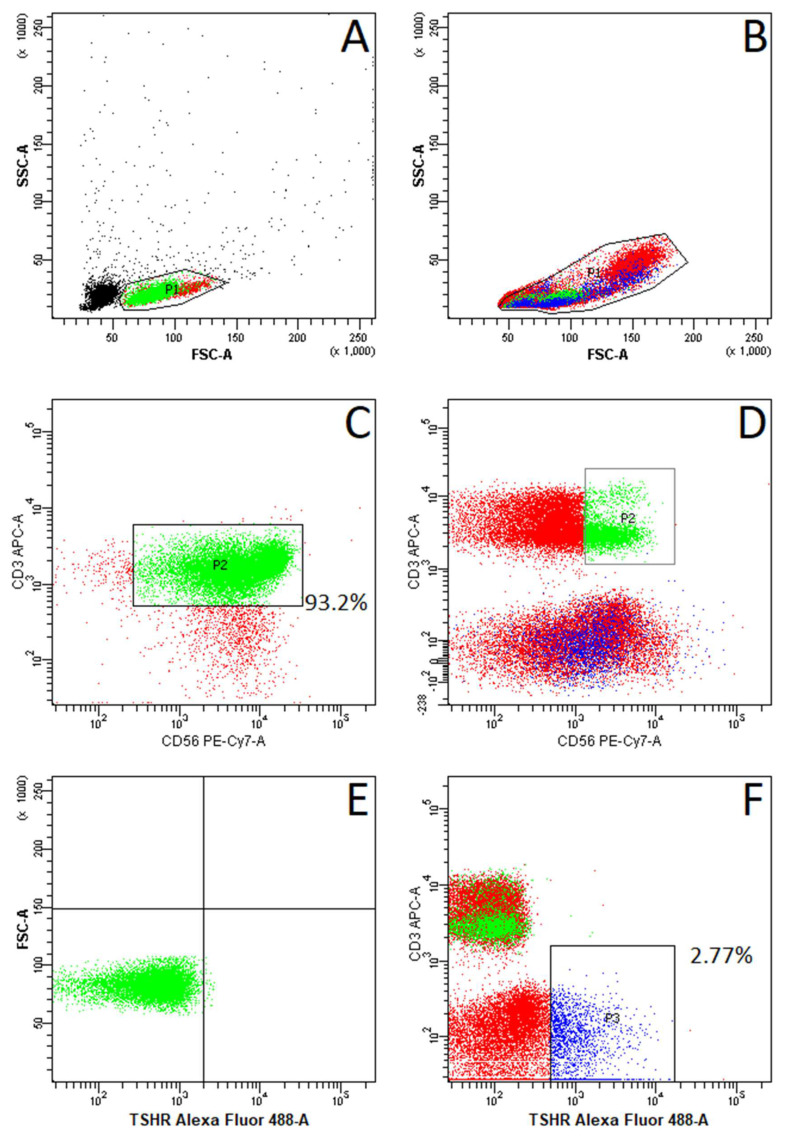
Exemplary plots of flow cytometry fluorescence-activated cell sorting (FACS) analysis showing the gating strategy of analyzed cells: (**A**): Natural Killer T (NKT) cells after magnetic sorting. (**B**): Peripheral blood mononuclear cells (PBMCs). (**C**): CD3+ CD56+ NKT cells after sorting. (**D**): CD3+ CD56+ NKT cells (green) among PBMCs. (**E**): Thyroid-stimulating hormone receptor (TSHR)-expressing NKT cells after sorting. (**F**): TSHR-expressing cells among PBMCs (blue). Black dots represent particles not recognized as single cells. Red dots represent negatively gated cells.

**Figure 2:**
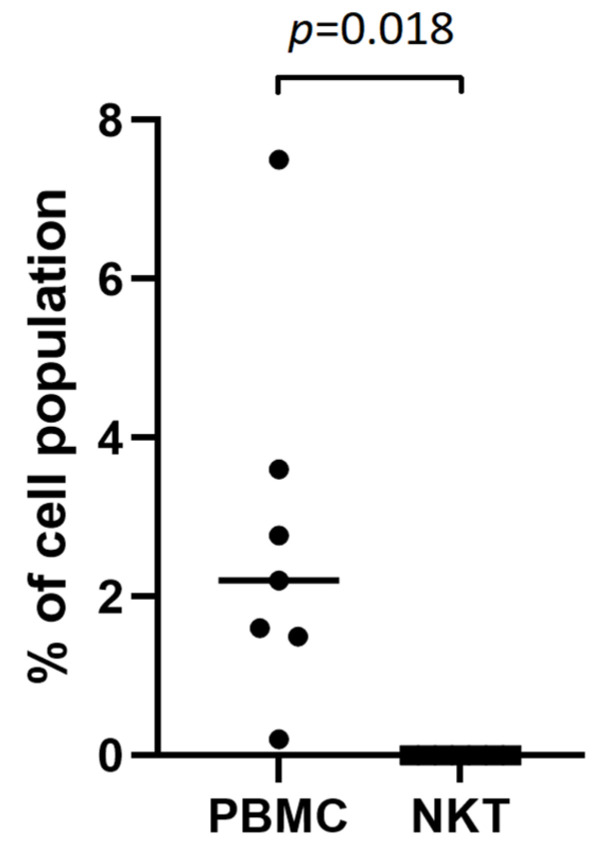
Percentage of cells expressing TSHR among whole PBMC fractions and in NKT cells.

FACS analysis of NKT cells revealed no TSHR+ NKT cells in any of the patients, showing only neglectable detection of high-autofluorescence individual cells, analogous to the isotype control (Figure 1E). However, positive control staining conducted on seven patients detected TSHR+ cells (2.77%) among the total population of PBMCs (Figure 1F). The difference was statistically significant with *p* = 0.018 (Figure 2).

**Figure 3:**
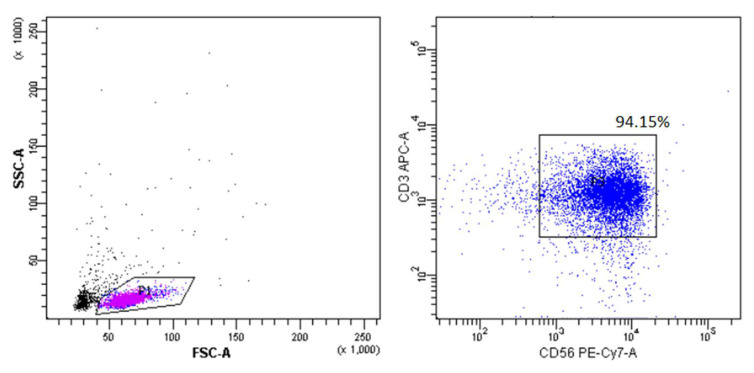
Gating strategy showing the purity of NKT cells isolated from buffy coats.

For further confirmation of the obtained results, a more sensitive method of RT-PCR was used in NKT cells isolated from healthy individuals. To achieve high RNA concentrations, NKT cells were isolated from up to 1 × 10^8^ PBMCs isolated from 10 buffy coats collected from healthy donors. The purity of the NKT cells after magnetic sorting—prior to RNA isolation—was measured by FACS and showed a high (median 94.15%) percentage of NKT cells (Figure 3). During the analysis, one result was excluded due to low amplification of the tested genes.

**Figure 4:**
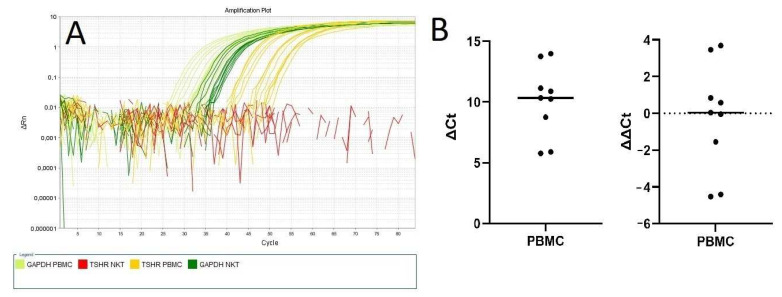
Reverse-transcription polymerase chain reaction (RT-PCR) results: (**A**): Amplification plots of target genes—(glyceraldehyde-3-phosphate dehydrogenase) *GAPDH* and *TSHR* from PBMC and NKT cells. (**B**): All measured ΔCt (relative TSHR gene expression to housekeeping gene expression) and ΔΔCt (ΔCt normalized to ΔCt median) values for *TSHR* gene in PBMC cells. Each dot identifies the mean expression calculated from triplets.

RT-PCR analysis has demonstrated no *TSHR* expression in NKT cells, while *TSHR* expression was present in the total PBMC population (Figure 4A). The results were normalized as positive (1) vs. negative (0) and significant difference was confirmed with Fisher’s exact test with *p* < 0.00001, and with chi-square test with *p* = 0.0002. The median value of ΔCt (relative *TSHR* gene expression to housekeeping gene expression) for the *TSHR* gene in PBMCs was 10.3 (Figure 4B).

## 3. Discussion

This study provides the first evidence of the absence of TSHR on the surface of NKT cells in the peripheral blood of both healthy individuals and patients with benign thyroid nodular disease with and without AITD. The use of both FACS and RT-PCR strengthens the validity of our findings. FACS provides a robust method for detecting surface proteins, while RT-PCR allows for the detection of mRNA expression, confirming the absence of TSHR at both the protein and gene levels in NKT cells. The absence of TSHR expression on NKT cells in both AITD and non-AITD patients suggests that NKT cells do not participate directly in the TSHR-mediated pathways involved in thyroid autoimmunity. This finding is significant as it challenges previous assumptions about the involvement of these immune cells in AITD pathophysiology [5,27,28,29]. However, this does not exclude the involvement of NKT cells in AITD development driven through indirect mechanisms involving other humoral, cellular, or hormonal factors and further modulated by already triggered pathological processes. Additionally, NKT cells are suggested to play a role in AITD development and progression at the local thyroid gland tissue level, through the switch of activating versus inhibiting regulatory roles [29].

Despite the lack of TSHR on NKT cells, the relationship between peripheral blood NKT cell quantity and TSH levels has been previously documented. Adamczewski et al. [4] showed a significant increase in NKT cells after in vivo administration of rhTSH in patients after total thyroidectomy due to differentiated thyroid cancer. Moreover, Miko et al. [5] found a remarkably elevated peripheral NKT level in euthyroid and subclinical hypothyroid women with thyroid autoimmunity experiencing reproductive failure.

The detection of TSHR expression in the total PBMC population, but not in isolated NKT cells, suggests that TSHR expression on immune cells might be restricted to specific PBMC subpopulations. This aligns with previous studies identifying a high expression of TSHR mainly on DCs and B cells but a low expression on T cells or NK cells [2,3]. As mentioned above, NKT cells may be affected by abnormal TSH levels through complex, indirect interactions with other immune cells. One potential intermediary mechanism involving NKT cells in AITD pathophysiology may be related to interactions with antigen-presenting cells (APCs). APCs can capture and transform antigens in order to present them within the context of appropriate major histocompatibility complex (MHC) molecules [30]. NKT cells have a diverse range of TCRs, allowing them to recognize various antigens, though CD1d-restricted invariant NKT (iNKT) cells specifically recognize lipid antigens presented by CD1d molecules [31]. CD1d is a non-polymorphic, MHC class I-like molecule expressed on various APCs, primarily DCs, and to a lesser extent on B cells and macrophages. Its primary function is to present lipid antigens, such as glycolipids and glycosphingolipids, to NKT cells. The interaction between CD1d and the TCR on NKT cells is crucial for their activation. Upon recognizing lipid antigens presented by CD1d, NKT cells rapidly produce a diverse array of cytokines, such as interferon gamma (IFN-γ), interleukin-4 (IL-4), and interleukin-17 (IL-17), modulating the immune response. This activation triggers both direct cytotoxic effects and the recruitment and activation of other immune cells, thereby playing a pivotal role in bridging innate and adaptive immunity. The ability of CD1d to present both self and foreign lipid antigens allows NKT cells to participate in immune surveillance and regulation, contributing to the body’s defense mechanisms against infections, tumor surveillance, and the modulation of autoimmune responses [32,33].

A role of DCs, as a population of the most potent APCs, should be considered in the TSH-mediated regulation of NKT cell function. In humans, two main DC groups are known: conventional/myeloid (cDCs) and plasmacytoid (pDCs) [34,35]. Through the expression of numerous co-stimulatory molecules and a unique secretion profile, DCs are involved in cross-interactions with other immune cells. Thus, DCs play a role in stimulating the immune response and ensuring immunological tolerance, making them key subjects in research on the pathogenesis of autoimmune diseases [36]. Interestingly, only a few studies assess DCs’ role in human AITD. Leskela et al. [37] revealed a notably elevated amount of pDCs in thyroid tissue compared to peripheral blood in patients with GD and HT. Stasiołek et al. [38] observed a significantly higher percentage of cDCs in fine-needle aspiration biopsy (FNAB) material than in peripheral blood in a group of AITD patients. Other studies also revealed an increased DC population in thyroid-infiltrating cells in patients with both GD and HT [39,40,41]. Elevated NKT [5,29] and DC [37,38,39,40,41] levels in AITD patients suggest a complex, indirect, co-stimulatory interaction between NKT cells and DCs as a possible mechanism involving NKT cells in thyroid diseases.

Another potential mechanism involving TSH in correlation with peripheral NKT cells was also postulated. Studies by Bessoles et al. [42] highlighted the complex interaction between glycolipids and environmental signals in regulating cytokine production by NKT cells, thereby modulating immune responses. To elucidate NKT cells’ regulatory mechanisms, their activation by interleukin-2 (IL-2) and associated signaling pathways were examined. IL-2 uniquely activates the Signal Transducer and Activator of Transcription 6 (STAT6) pathway in NKT cells, leading to the production of both pro-inflammatory (IFN-γ) and anti-inflammatory (IL-4) cytokines, and to cell proliferation.

Clinical studies by Komorowski et al. [43,44] indicated that patients with primary hypothyroidism and elevated TSH levels exhibited increased IL-2 concentrations in peripheral blood, suggesting that elevated TSH levels may influence IL-2 production. Hence, it can be hypothesized that TSH might modulate NKT cell function indirectly through IL-2 mediation, thereby affecting the immune response. This linkage provides a potential pathway wherein TSH could exert its effects on NKT cells via IL-2, underscoring a novel intersection between endocrine and immune regulation.

This study has potential limitations as we used only two methods for the detection of TSHR expression on NKT cells. The FACS method has been broadly accepted for the detection of TSHR on cells of non-thyroid origin including peripheral blood cells [45,46,47]. Anti-TSHR antibody (clone C-10) was already proven to sufficiently detect TSHR [48,49,50,51]. Additionally, we confirmed that Alexa Fluor-conjugated antibody was sufficient for detecting even low TSHR expression (Figure 1F). However, we are aware that the FACS method is not sufficient to prove the absence of expression of any receptor. Therefore, our findings were verified by a very sensitive method of RT-PCR, which is considered a gold standard in gene expression analysis, including TSHR expression in immune cells [52,53]. Nevertheless, we believe that our results require further confirmation in studies with the application of other methods, including those with the highest available sensitivity (e.g., next-generation sequencing, NGS).

## 4. Materials and Methods

### 4.1. Patients

This study involved 86 patients with cytologically benign thyroid nodules (68 and 18 females and males, respectively) treated in the Department of Endocrinology and Metabolic Diseases, Polish Mother’s Memorial Hospital—Research Institute, Łódź, Poland. Among the study group, 28 patients were diagnosed with AITD, including 7 cases of Hashimoto’s thyroiditis treated with L-thyroxine, 14 cases of euthyroid chronic thyroiditis, and 7 cases of Graves’ disease, with 5 patients treated with thiamazole and 2 euthyroid patients who had received radioiodine therapy. The mean age of the study participants was 59.7 ± 13.69 years.

### 4.2. Inclusion Criteria

AITD diagnosis was based on standard criteria including elevated thyroid peroxidase antibody (anti-TPO) level and/or elevated thyroglobulin antibody (anti-Tg) level, or elevated TSH receptor antibody (TRAb) level [54].

### 4.3. Biochemical Analysis

Serum levels of TSH, free triiodothyronine (FT3), free thyroxine (FT4), anti-Tg, anti-TPO, and TRAb were measured by the electrochemiluminescence immunoassay (ECLIA) with Cobas e601 analyzer (Roche Diagnostics, Indianapolis, IN, USA).

### 4.4. NKT Cell Isolation

Peripheral blood samples (2 × 4.9 mL) were collected from each patient via venipuncture into EDTA-containing Blood Collecting Systems (Sarstedt, Nümbrecht, Germany). Peripheral blood mononuclear cells (PBMCs) were isolated by gradient centrifugation at 400 g for 30 min using Histopaque^®^-1077 (Thermo Fisher Scientific, Waltham, MA, USA). NKT cells were then isolated from PBMCs using a CD3+ CD56+ NKT Cell isolation kit with a magnetic bead cell separator (Miltenyi Biotec, Bergisch Gladbach, Germany). In each patient, the cell purity was measured and the median NKT cell percentage was 93.2% (range 99.1–80.1%). NKT cells were gated as CD3+ CD56+ cells for further analysis.

### 4.5. Fluorescence-Activated Cell Sorting (FACS)

To analyze TSHR expression on NKT cells, fluorescence-activated cell sorting (FACS) was employed. Fluorochrome-conjugated antibodies against human CD3 (Becton Dickinson, Franklin Lakes, NJ, USA) and CD56 (Becton Dickinson, NJ, USA) were used to identify NKT cells, and an antibody against human TSHR (Santa Cruz, Santa Cruz, CA, USA) was used to determine TSHR expression on isolated NKT cells. Additional positive staining for antibody validation was conducted on whole blood samples from 7 patients (including 3 patients with AITD and 4 patients without AITD) to recognize TSHR+ cells in the PBMC population (Figure 1A–D). Appropriate isotype controls were used. FACS analyses were conducted using a BD FACSCanto II flow cytometer (Becton Dickinson, NJ, USA).

### 4.6. Reverse-Transcription Polymerase Chain Reaction (RT-PCR)

In order to confirm the obtained results with the application of a different method, TSHR expression was analyzed by reverse-transcription polymerase chain reaction (RT-PCR). To obtain a high number of NKT cells (0.5–1 × 10^6^), needed for sufficient RNA isolation, up to 100 mL of peripheral blood would have been needed from each patient. Therefore, for ethical reasons, we obtained a 10 buffy coats from the healthy blood donors registered in the Regional Center of Blood Donation and Treatment. PBMCs and NKT cells were isolated as described above, using a CD3+ CD56+ NKT Cell isolation kit with a magnetic bead cell separator (Miltenyi Biotec, Germany). Total ribonucleic acid (RNA) was extracted from both PBMCs and NKT cells, and *TSHR* expression was analyzed by RT-PCR using TaqMan assays. The *GAPDH* gene was used as a housekeeping gene. Analyses were performed on a 7500 Real-Time PCR System (Thermo Fisher Scientific, USA). Each measurement was conducted in triplets.

### 4.7. Statistical Analysis

Descriptive statistics of the collected material contained the mean and standard deviation (SD). For comparisons between the groups, Student’s *t*-test for normally distributed variables and the Mann–Whitney U test for the other ones were used. Positive/negative values were tested using Fisher’s exact test as well as with the chi-square test with Yates correction. The normality of data distributions was verified by the Shapiro–Wilk test. In all the tests, *p*-value < 0.05 was considered significant. Statistical Package for the Social Sciences (SPSS 20.0) software for Windows was used for all the calculations.

### 4.8. Ethics Procedures

Written informed consent was obtained from all patients for the procedures performed after their purpose and course were thoroughly explained. This study was approved by the Ethics Committee of the Polish Mother’s Memorial Hospital—Research Institute, Lodz, Poland (approval code—41/2021).

## 5. Conclusions

The present study demonstrated for the first time that TSHR expression was not found on NKT cells. This observation provides new insight on the potential mechanism of TSH’s impact on NKT cells, which seems to be indirect. These findings have significant implications as therapeutic strategies targeting TSHR on immune cells should consider the specific immune cell subtypes involved in the disease development mechanisms. As the expression of TSHR on NKT cells had not been studied before, our findings require further confirmation with the application of different methods.

## Figures and Tables

**Table 1:** Clinical characteristics of the study group.

	Patients with AITD Mean ± SD (n = 28)	Patients without AITD Mean ± SD (n = 58)	*p* Value (AITD Versus Non-AITD Group)
Age	55.54 ± 11.4	61.72 ± 14.43	*p* = 0.05
Female/Male (n)	23/5	45/13	*p* = 0.63
fT4 (0.93–1.7 ng/dL)	1.25 ± 0.27	1.27 ± 0.34	*p* = 0.9
fT3 (2–4.4 pg/mL)	2.83 ± 1.01	2.94 ± 0.69	*p* = 0.56
TSH (0.27–4.2 uIU/mL)	1.6 ± 2.39	1.3 ± 1.1	*p* = 0.42
anti-TPO (<34 IU/mL)	157.28 ± 149.04	11.39 ± 4.9	*p* < 0.05 *
anti-Tg (<115 IU/mL)	187.37 ± 193.65	15.5 ± 8.12	*p* < 0.05 *
TRAb (0.8–1.75 IU/L)	2.63 ± 5.17	0.99 ± 0.26	*p* = 0.09

Abbreviations: fT4, free thyroxine; fT3, free triiodothyronine; TSH, thyroid-stimulating hormone; anti-TPO, thyroid peroxidase antibodies; anti-Tg, thyroid antithyroglobulin antibodies; TRAb, thyroid-stimulating hormone receptor antibodies. * indicates statistical significance.

## Data Availability

The source data are available on reasonable request from the corresponding author.

## Abbreviations

AITD	autoimmune thyroid disease
Anti-Tg	thyroid antithyroglobulin antibodies
Anti-TPO	thyroid peroxidase antibodies
APCs	antigen-presenting cells
CD	cluster of differentiation
DCs	dendritic cells
pDCs	plasmocytoid dendritic cells
cDCs	conventional dendritic cells
EDTA	ethylenediamine tetraacetic acid
FACS	fluorescence-activated cell sorting
FNAB	fine-needle aspiration biopsy
GAPDH	glyceraldehyde-3-phosphate dehydrogenase
GD	Graves’s disease
HT	Hashimoto’s thyroiditis
IFN-y	interferon-gamma
IL-2	interleukin-2
IL-4	interleukin-4
IL-17	interleukin-17
iNKT	invariant Natural Killer T cells
MCH	major histocompatibility complex
NK	Natural Killer cells
NKT	Natural Killer T cells
NIS	sodium/iodide symporter
PBMCs	peripheral blood mononuclear cells
mRNA	messenger ribonucleic acid
rhTSH	recombinant human thyroid stimulating hormone
RNA	ribonucleic acid
RT-PCR	reverse-transcription polymerase chain reaction
SNP	single-nucleotide polymorphism
STAT6	Signal Transducer and Activator of Transcription 6
TCR	T-cell receptor
Tg	thyroglobulin
TPO	thyroid peroxidase
TRAb	thyroid-stimulating hormone receptor antibodies
TSH	thyroid-stimulating hormone
TSHR	thyroid-stimulating hormone receptor
